# Natural History of Rare Tumor Risk Syndromes: A Scoping Review to Inform Cost-Effectiveness Models

**DOI:** 10.36469/001c.167564

**Published:** 2026-10-12

**Authors:** Carolina Di Fabrizio, Judite Gonçalves, Zillur R. Shabuz, Andrew Titman, Paula Boaventura, Svetlana Bajalica Lagercrantz, Barbara Peleteiro, Sara Pereira, Liliana Sousa, Stefan Aretz, Paula Soares, Janneke Schuurs Hoeijmakers, Jean Christophe Thery, Hildegunn Hoberg Vetti, Judith Balmaña, Joan Brunet, Carla Oliveira, Céu Mateus, PREVENTABLE Consortium

**Affiliations:** 1 NOVA School of Business and Economics, NOVA University of Lisbon, Carcavelos, Portugal; 2 Lancaster School of Mathematical Sciences, Lancaster University, Bailrigg, Lancaster, UK; 3 School of Mathematical Sciences, Lancaster University, Bailrigg, Lancaster, UK; 4 i3S – Instituto de Investigação e Inovação em Saúde, Universidade do Porto, Porto, Portugal; 5 Karolinska Institutet, Department of Oncology-Pathology, Stockholm, Sweden; 6 Hospital Epidemiology Center, Unidade Local de Saúde São João, Porto, Portugal; 7 ERN GENTURIS - European Reference Network on Genetic Tumour Risk Syndromes, Nijmegen, The Netherlands; 8 Institute of Human Genetics, Medical Faculty, University of Bonn, Bonn, Germany; 9 Institute of Molecular Pathology and Immunology of the University of Porto (Ipatimup), Porto, Portugal; 10 Faculty of Medicine of the University of Porto (FMUP), Porto, Portugal; 11 Department of Human Genetics, Expert Center for PHTS Department of Human Genetics, Expert Center for PHTS, Radboud University Medical Center, Nijmegen, The Netherlands; 12 Department of Medical Oncology Université Rouen Normandie, Normandie Université, Inserm U1245, FHU G4 genomics, Centre Henri Becquerel, Rouen, France; 13 Western Norway Familial Cancer Center, Department of Medical Genetics, Haukeland University Hospital, Bergen, Norway; 14 Faculty of Health Studies, VID Specialized University, Bergen, Norway; 15 Hereditary Cancer Genetics, Vall d’Hebron Institute of Oncology (VHIO), Barcelona, Spain; 16 Medical Oncology Department, Vall d’Hebron Barcelona Hospital Campus, Barcelona, Spain; 17 Hereditary Cancer Program, Catalan Institute of Oncology (ICO) and IDIBGI, Girona, Spain; 18 Division of Health Research, Lancaster University, Bailrigg, Lancaster, UK

**Keywords:** rare diseases, hereditary cancer syndromes, cancer surveillance, natural history, decision-analytic modeling, cost-effectiveness analysis

## Abstract

**Background:**

Hereditary cancer syndromes account for approximately 10% of all cancers. Many of these, classified as rare tumor risk syndromes (RTRS), lack comprehensive data on natural history, lifetime cancer risk, and the long-term impact of surveillance. These evidence gaps limit the development of decision-analytic disease progression models and cost-effectiveness analyses needed to support healthcare decision-making.

**Objective:**

To map the available evidence on the natural history, cancer risk, and surveillance strategies in selected RTRS to inform disease progression models and future economic evaluations.

**Methods:**

A scoping review was conducted on 8 RTRS: Birt-Hogg-Dubé syndrome, familial gastrointestinal stromal tumor, familial melanoma, hereditary diffuse gastric cancer, hereditary leiomyomatosis and renal cell cancer, Li-Fraumeni syndrome, Peutz-Jeghers syndrome, and PTEN hamartoma tumor syndrome. Systematic searches of PubMed, Embase, and relevant gray literature sources were performed between April and June 2026 for studies published since 2010.

**Results:**

After screening 16 631 records, 80 studies were included in the review. Findings indicate significant heterogeneity in data availability and surveillance effectiveness. Some RTRS have relatively well-defined surveillance recommendations, whereas others remain poorly characterized. Across studies, cancer incidence was markedly higher and occurred earlier than in the general population, with lifetime cancer risks approaching 100% in certain syndromes. However, only 44% of studies reported follow-up protocols and fewer than 12% compared surveillance with expectant management. Limited data on postdiagnosis survival further complicates economic evaluations of alternative care strategies.

**Conclusions:**

The evidence base for RTRS is heterogeneous, particularly regarding longitudinal outcomes and comparative surveillance strategies. These limitations restrict the parameterization of disease progression models and the evaluation of the cost-effectiveness of surveillance strategies, leaving substantial uncertainty about optimal management strategies. Significant evidence gaps remain in the natural history and management of RTRS. This review provides a foundation for future cost-effectiveness analyses by mapping existing evidence and identifying critical knowledge gaps in RTRS care.

## BACKGROUND

In 2020, there were 18 million cases of cancer worldwide and 10 million deaths attributable to the disease. Moreover, it is estimated that between 2020 and 2050, cancer will have an economic cost of approximately US $25.2 trillion.^1^ Despite being underdiagnosed, hereditary cancer syndromes account for nearly 10% of cancers. To date, more than 40 distinct types of hereditary cancer syndromes have been identified.^2^ Most are rare, affecting 5 per 10 000 people or less, and are known as rare tumor risk syndromes (RTRS).

Although individually considered rare, together RTRS encompass a significant number of patients who face common challenges: delayed diagnosis, limited preventive measures for both patients and their relatives, and therapeutic mismanagement, often requiring proactive care of a patient’s family members and complex patient education for the whole kindred.^2,3^

Individuals with hereditary cancer risk syndromes carry pathogenic germline genetic variants usually passed down from one or both parents, which strongly predispose them to the development of malignant tumors, sometimes with onset during childhood.^4^ Patients diagnosed with some RTRS have up to 100% lifetime risk of developing cancer.^5^ Intensive surveillance and risk-reduction strategies for asymptomatic RTRS patients are a key approach to safeguarding the supreme interest of those individuals and their families.^6^ Implementation of cancer risk-reduction measures in asymptomatic RTRS patients may increase their life expectancy and healthy aging. Furthermore, individuals diagnosed at an earlier stage are more likely to achieve better survival and to significantly benefit from better care experiences, reduced treatment-related complications, and enhanced quality of life in comparison to those diagnosed at a later stage.^7^

Although there is an abundance of information regarding diagnosis,^8,9^ management,^10–12^ and treatment,^13^ data on the natural history of certain RTRS, necessary for cost-effectiveness of alternative treatment/management strategies and other types of studies, are lacking. Moreover, many studies on clinical surveillance strategies for these syndromes have been published only recently, and the information therein has not been compiled and synthetized. This scoping review addresses these gaps by documenting the natural history, cancer incidence, and cancer risk of the following RTRS: Birt-Hogg-Dubé syndrome (BHD), familial gastrointestinal stromal tumor (GIST), familial melanoma (FM), hereditary diffuse gastric cancer (HDGC), hereditary leiomyomatosis and renal cell cancer (HLRCC), Li-Fraumeni syndrome (LFS), Peutz-Jeghers syndrome (PJS), and *PTEN* hamartoma tumor syndrome (PHTS). Important information regarding surveillance programs is also covered.

The selection of these eight RTRS was based on two factors. First, information regarding these syndromes is scarce, highlighting the need for a comprehensive literature review. Second, this study is part of a larger research initiative, the PREVENTABLE project,^14^ funded by Horizon Europe, which focuses specifically on these syndromes to enhance understanding and improve management strategies.

## METHODS

This study is a scoping review, given that literature is sparse, scattered, and heterogeneous. Its aims were to map the range, nature, and extent of existing research; identify gaps; and provide a summary of the available information to inform the modeling of disease progression in RTRS patients, providing a basis for future economic evaluation or other studies. This review was conducted and reported in accordance with the Preferred Reporting Items for Systematic Reviews and Meta-Analyses extension for Scoping Reviews (PRISMA-ScR^15^) (**Appendix, Section 2**). No review protocol was prospectively registered or published. A formal critical appraisal of the included evidence was not undertaken, as the objective of the review was to map and characterize the available evidence rather than to assess its methodological quality.

### Data Sources and Search Strategy

Literature searches were performed between April and June 2026. Two major databases, one medical (PubMed) and one general (Embase) were searched to identify relevant studies about the eight RTRS, their natural history, risk of developing different cancers, and surveillance programs (if they were applied in the study and not simply a recommendation). Gray literature was also searched through ClinicalTrials.gov and relevant institutional and professional websites, including ERN GENTURIS, Orphanet, the National Cancer Institute, and the European Society for Medical Oncology. Disease registries and syndrome-specific resources such as PHTS Patient Registry UK and BHD Foundation were also searched. Searches were focused on patients with RTRS, both symptomatic and asymptomatic carriers of a germline pathogenic or likely pathogenic variant of the affected genes. Search terms included alternative names of the syndromes, proteins or genes involved, combined with Boolean operators.

Only studies involving human beings, written in English, Portuguese, or Spanish were included. Considering the identification of numerous genes implicated in these syndromes, advancements in technology and genetic diagnostics, and to ensure the compilation of the most up-to-date evidence, only studies published from 2010 until the search date were taken into consideration. Studies without published results (eg, protocols), those regarding very specific treatments, diagnostic methods (such as genetic testing techniques) or features of the syndrome, single case/family reports or reviews/meta-analyses were excluded. Studies performed in vitro or in animals and nonprimary research studies (eg, commentaries or conference abstracts) were also not considered. The detailed search strategy and inclusion and exclusion criteria can be found in the **Appendix, Section 1**.

Database search was accompanied by hand searching of reference lists of included studies to identify any additional relevant studies. Reviews and conference abstracts, identified in the primary search but not selected for inclusion, were also reviewed for relevant studies cited therein.

No formal pilot testing of the screening criteria or data charting form was conducted. The predefined eligibility criteria and data fields were applied consistently throughout the review.

For the purposes of this review, cancers were grouped according to the affected organ, without distinguishing histological subtypes across syndromes. However, within each syndrome, the tumor type and histology typically associated with that syndrome were retained. Thus, “gastric cancer” may refer to different histological entities depending on the syndrome, including gastric adenocarcinoma, diffuse-type gastric cancer, or gastrointestinal stromal tumors.

### Study Selection

Results from database searches were imported into Covidence systematic review software (Veritas Health Innovation), and duplicates were removed using the software’s automated tools. Following deduplication, titles and abstracts were screened by a primary reviewer against predefined inclusion and exclusion criteria. Full texts of potentially eligible articles were subsequently retrieved and assessed for eligibility by the same reviewer. Any uncertainties were resolved through discussion with a second reviewer with modeling and economic evaluation expertise. To strengthen the robustness and clinical relevance of the selection process, the search strategy, preliminary study selection, and extracted evidence for each syndrome were reviewed by clinical experts from the corresponding syndrome-specific teams participating in the PREVENTABLE consortium. These experts provided feedback on study relevance, completeness of captured evidence, and alignment with real-world clinical pathways. Where appropriate, this consultation process led to refinements in study selection, data extraction and interpretation, while maintaining consistency with the predefined review objectives, eligibility criteria, and methodological procedures.

### Data Extraction and Synthesis

Data extraction was performed using a structured Microsoft Excel form developed specifically for this review. Extracted data included study characteristics (author, year, country, publication type, study duration, and number of families included); participant characteristics (sample size, age, sex, family history, and inclusion of symptomatic patients and/or asymptomatic carriers); and syndrome-related clinical variables. These comprised incidence at birth, age at syndrome diagnosis, age at first cancer diagnosis, relevant benign lesions, main cancer types, age at diagnosis, treatment and clinical course, sex-related differences, second malignant neoplasms, and cumulative cancer risk. Surveillance-related variables included the age at initiation and cessation of surveillance, surveillance frequency and modality, and cancers or other lesions detected through surveillance. Life expectancy and prenatal diagnosis were also extracted when reported.

The extracted variables were selected to inform key inputs for disease progression models and cost-effectiveness analyses, including transition probabilities (cumulative cancer incidence, age at onset), mortality parameters (postcancer survival, cancer-related death), and surveillance-related outcomes. Health state utility values were not extracted, as none of the included studies reported preference-based quality-of-life measures for these syndromes; this gap is discussed below.

Extraction was led by the primary reviewer, with extracted data for each syndrome subsequently reviewed and discussed with cost-effectiveness modeling and syndrome-specific clinical experts. These discussions focused on verifying the interpretation and contextualization of clinical outcomes for modeling purposes. Any uncertainties arising during data extraction were resolved through consensus between the lead reviewer and co-authors.

We performed literature searches, study selection, and data extraction separately for each of the eight syndromes to keep tasks manageable, following the methodology described above. Due to heterogeneity and the descriptive nature of the results, a narrative synthesis approach was used to present the results, complemented by summary tables of the included studies’ characteristics and clinical information (**Appendix, Section 4**).

## RESULTS

### Search Results

After exclusion of duplicates, 16 631 titles and abstracts from PubMed and Embase were screened, with initial results ranging from 466 (FM) to 7192 (PJS) studies. Of the studies ultimately included, 9 were identified through complementary gray literature and citation searches. At this stage, 16 023 studies were excluded, as it was clear from the title or abstract they did not meet the inclusion criteria. In total, 605 studies were eligible for full-text screening. Among these, 80 studies fulfilled the established criteria and were included in this review: 16 studies on PJS, 13 on HDGC, 12 on PHTS, 11 on LFS, 11 on BHD, 9 on FM, 7 on HLRCC, and 1 study on familial GIST. **Section 3** of the **Appendix** shows the PRISMA data charting tables for each syndrome.

### Study Characteristics

**Section 4** of the **Appendix** includes summary tables of the 80 included studies’ characteristics, grouped by syndrome.

Regarding geographic origin, studies were mainly from European (n = 41 [51.25%]: Denmark, France, Germany, Italy, Netherlands, Portugal, Spain, Sweden, Switzerland, United Kingdom) or American (n = 26 [32.5%]: Brazil, Canada, Uruguay, United States) countries, with lower representation of Asian countries (n = 13 [16.2%]; China, Japan, South Korea, Taiwan). The Netherlands alone contributed with 15 studies (18.75%). There were 12 (15.0%) multicentric studies.

**Figure 1.**
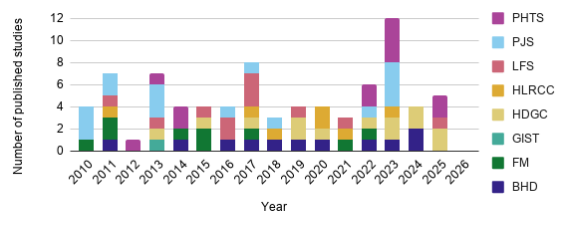
Time of Publication of Included Studies Abbreviations: BHD, Birt-Hogg-Dubé syndrome; FM, familial melanoma; GIST, gastrointestinal stromal tumor; HDGC, hereditary diffuse gastric cancer; HLRCC, hereditary leiomyomatosis and renal cell cancer; LFS, Li-Fraumeni syndrome; PHTS, *PTEN* hamartoma tumor syndrome; PJS, Peutz-Jeghers syndrome.

Most studies were published on or after 2018 (n = 41 [51.2%]), with only 12 studies (15%) published before 2013 (**Figure 1**).

The search yielded mostly retrospective cohort studies (n = 56 [70%]), followed by prospective studies (n = 21 [26.25%]), and the remaining 3 studies (3.75%) were case-controls. The duration of clinical follow-up varied substantially, ranging from a few months to up to 38 years.^16^ Outcome measures were predominantly collected by retrospective review of medical records (clinical, pathological, histological and imaging) and cancer registries.

### Patient Characteristics

All studies reported the number of patients included, with an average of 185 participants, ranging from 12^17^ to 883,^18^ and with a higher representation of females among participants (mean, 58.78%; range, 36.4%-100%). Every study included adult patients in their cohort and at least one study per syndrome included patients under 18 years old (**Appendix, Section 4**). There was considerable heterogeneity in the clinical status of participants across studies. Of the 80 included studies, 50 enrolled only symptomatic individuals, 9 included only asymptomatic carriers of the pathogenic variant, and 21 reported on both symptomatic and asymptomatic patients. Notably, this variation was observed across all eight syndromes, with no syndrome having a fully homogeneous study population.

### RTRS Prevalence

Data published in the literature point toward a very low prevalence of each of the eight syndromes, with fewer than 5 cases per 10 000 people.^19–23^

Only 5 studies, pertaining to 3 syndromes (BHD, HLRCC, and PJS) reported the estimated prevalence based on their own data. Two other studies^24,25^ reported the estimated prevalence of the allelic variant (a specific variant in a gene sequence) in their specific study population, with no details on the prevalence of the syndrome. **Table 1** details prevalence data and affected genes and proteins.

**Table 1.** Reported Specific Prevalences, Main Affected Genes and Encoding Proteins, by Rare Tumor Risk Syndrome

**Syndrome**	**Prevalence/Frequency**	**Affected Genes**	**Encoding Protein**
BHD	0.0567/100 000^26^	*FCLN*	Foliculin
FM	NR	*CDKN2A, CDK4*	p16^INK4^ and p14^ARF^, cyclin-dependent kinase 4
GIST	NR	*KIT, PDGFRA*	Receptor tyrosine kinases
HDGC	NR (Frequency of CDH1 variant allele in general population: 100/100 000)^24^	*CDH1*	E-cadherin
HLRCC	6.70/100 000^27^	*FH*	Fumarate hydratase
LFS	NR (Prevalence of the mutation in newborns in Parana, Brazil: 270/100 000)^25^	*TP53*	P53
PJS	0.55/100 000 (range, 0.51-0.60/100 000)^28–30^	*STK11*	Serine threonine kinase 11
PHTS	NR	*PTEN*	*PTEN*

Abbreviations: BHD, Birt-Hogg-Dubé; CDH1, cadherin 1; CDK4, cyclin-dependent kinase 4; CDKN2A, cyclin-dependent kinase inhibitor 2A; FH, fumarate hydratase; FCLN, folliculin; FM, familial melanoma; GIST, gastrointestinal stromal tumor; HDGC, hereditary diffuse gastric cancer; HLRCC, hereditary leiomyomatosis and renal cell cancer; LFS, Li-Fraumeni syndrome; NR, not reported; PDGFRA, platelet-derived growth factor receptor alpha; PHTS, *PTEN* hamartoma tumor syndrome; *PTEN*, phosphatase and tensin homolog; PJS, Peutz-Jeghers syndrome; STK11, serine/threonine kinase 11; TP53, tumor protein 53.

### Age at RTRS Diagnosis and Age at First Tumor

Most studies reported age at first tumor (n = 45 [56%]), with fewer studies (n = 25 [31%]) reporting age at RTRS (genetic) diagnosis. The only study on familial GIST in this review^31^ does not clarify if the age of diagnosis specified corresponds to age of RTRS diagnosis or age of gastric cancer diagnosis.

While tumors in LFS, HLRCC, FM, and PHTS may affect children and manifest during the first or second decade of life, the other RTRS considered here typically present their first tumor after the age of 30 or 40. Adrenocortical carcinoma, in LFS, is the type of cancer that affects the youngest patients, with a median age at diagnosis of 3 years old.^32^

In several cases, diagnosis of hereditary cancer syndrome occurred years after the onset of initial symptoms or even after the diagnosis of a first tumor. For example, individuals with HLRCC may develop benign cutaneous lesions approximately 10 years before the syndrome is diagnosed, and in a substantial proportion of patients, renal cell carcinoma is diagnosed before HLRCC itself.^33–35^ Similarly, Hu et al^36^ reported a mean interval of 7.6 years between symptom onset and BHD diagnosis.

Additional data on age at diagnosis were retrieved for specific syndromes. For example, in PHTS, second malignant neoplasia occurred most frequently in the sixth decade of life, although reported ages ranged from 21 to 71 years.

There was a strong tendency for RTRS carriers to develop cancer at a significantly younger age than in the general population: While mean age at melanoma diagnosis in the general population is 66 years old,^37^ patients affected by FM are diagnosed at a mean age of 38 to 39 years old, more than 25 years earlier.^38–40^ Similar differences can be observed in HDGC; Xicola et al^24^ have shown that mean age (±SD) at gastric cancer diagnosis in those patients was 42 (±16) years vs 68 for the general population.

RTRS carriers not only develop cancer at younger ages but also develop benign lesions that may require invasive treatments. Multiple studies^27,33,35,41,42^ showed that over 70% of women affected by HLRCC undergo hysterectomies in their 30s, while the same surgery is typically performed in women aged 45 to 49 years old in the general population.^43^

### Cancer Cumulative Incidence

**Figure 2.**
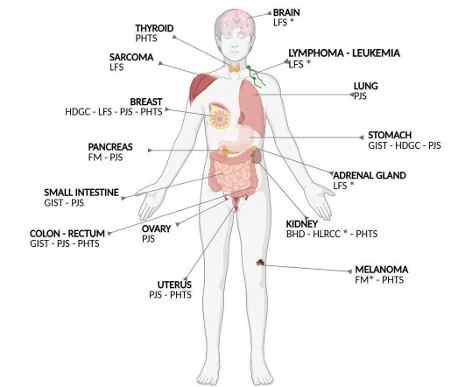
Main Organs Compromised by the Different Rare Tumor Risk Syndromes Abbreviations: BHD, Birt-Hogg-Dubé syndrome; FM, familial melanoma; GIST, gastrointestinal stromal tumor; HDGC, hereditary diffuse gastric cancer; HLRCC, hereditary leiomyomatosis and renal cell cancer; LFS, Li-Fraumeni syndrome; PHTS, *PTEN* hamartoma tumor syndrome; PJS, Peutz-Jeghers syndrome. *Both childhood and adult cancer.

Data on cancer incidence was available in 63 of 80 studies included. Additionally, treatment information was present in 31 of the 80 studies (39%) covering all RTRS. The most reported therapeutic options under use were chemotherapy^31,44,45^ and surgery.^17,27,35,46–56^ **Figure 2** shows the main organs compromised by the eight RTRS in both children and adults.

The breast can be affected by cancer in four different RTRS considered here: HDGC, LFS, PJS, and PHTS. Kidney, colon-rectum and stomach can be potentially compromised in three RTRS each. In the case of gastric cancer, while LFS and PJS patients may develop gastric adenocarcinoma, HDGC patients will have diffuse gastric cancer, and familial GIST individuals will be affected with tumors of the gastrointestinal stroma. Regarding renal cancer, incidence rates vary from 4.1% (PHTS) to 23.1% (HLRCC) and up to 43.8% for participants with BHD.

Among female patients, breast cancer was the most frequently reported malignancy, ranging from 7% (PJS) to 44% (PHTS),^57^ while the uterus might be compromised in both PJS and PHTS patients. Hendrix et al^58^ reported a higher breast cancer frequency of 72%; however, their cohort included only patients with PHTS who had already developed cancer and did not include asymptomatic carriers.

During childhood, patients may develop brain cancer (LFS, 20%), adrenocortical carcinoma (LFS, 41%), sarcoma (LFS: osteosarcoma, 30%; soft tissue sarcoma, 23%^59^), melanoma,^16^ and renal cancer.^27,41^

For some RTRS, some studies reported gender differences. Female carriers of PHTS were more likely to develop, in addition to cancers affecting female-specific organs, thyroid and renal cancers and second malignant neoplasms,^57,60,61^ whereas men showed a higher incidence of colorectal cancer. Women also had higher mortality than men^44^ and a younger mean age at first cancer diagnosis.^58^

In LFS patients, De Andrade^62^ and Mai^46^ reported higher risk of death after a first cancer (but before a second malignant neoplasm) in men than in women. However, no gender differences have been described in other syndromes, such as FM,^63^ where both genders were considered to have the same risk. While breast cancer predominantly affected women in the RTRS included in this review, no cases of male breast cancer were identified, while 1 in 3 women with PHTS were reported to have bilateral breast cancer.^57,60,61^

Multiple primary tumors were also observed across syndromes. In familial melanoma, a mean of 40% of patients had a personal history of at least two malignant melanomas.^16,38,63–65^ Among patients with PHTS-related cancer, 24% developed two distinct cancer types and 5% developed three.^44^ **Table 2** reports all information retrieved on cancer incidence.

**Table 2.** Lifetime Cancer Cumulative Incidence in the Main Organs Affected by the Rare Tumor Risk Syndromes

**Syndrome**	**Percentage of Studies’ Population (Range Across Studies)**															
**Any CA**	**Breast^a^**	**CRC**	**Stomach**	**Kidney**	**Melanoma**	**Uterus**	**Pancreas**	**Sarcoma**	**SI**	**Ovary**	**Lung**	**Lymphoma/Leukemia**	**Brain**	**Thyroid**	**ACC**	
BHD^17,66–70^	NR	NA	NA	NA	4-25	NA	NA	NA	NA	NA	NA	NA	NA	NA	NA	NA
Familial GIST^31^	NR	NA	22	33	NA	NA	NA	NA	NA	33	NA	NA	NA	NA	NA	NA
FM^16,38,40,64,65,71^	NR	NA	NA	NA	NA	53-100	NA	7-23	NA	NA	NA	NA	NA	NA	NA	NA
HDGC^48–51,72–74^	NR	8-31	NA	22-61	NA	NA	NA	NA	NA	NA	NA	NA	NA	NA	NA	NA
HLRCC^27,33–35,41,42,75^	NR	NA	NA	NA	6-92	NA	NR	NA	NA	NA	NA	NA	NA	NA	NA	NA
LFS^5,25,32,46,59,62,76–79^	7-100	9-79	NA	NA	NA	NA	NA	NA	2-30	NA	NA	NA	3-12	2-13	NA	2-23
PJS^28–30,47,53,54,80–87^	8-67	1-16	1-13	1-3	NA	NA	4 (C)	1-6	NA	1-7	1-11	1-13	NA	NA	NA	NA
PHTS^57,60,61,88–93^	20-60	16-45	1-6	NA	1-7	1-6	2-14 (E)	NA	NA	NA	NA	NA	NA	NA	1-29	NA

Abbreviations: ACC, adrenocortical carcinoma; BHD, Birt-Hogg-Dubé syndrome; C, cervix; CA, cancer; CRC, colorectal cancer; E, endometrium; FM, familial melanoma; GIST, gastrointestinal stromal tumor; HDGC, hereditary diffuse gastric cancer; HLRCC, hereditary leiomyomatosis and renal cell cancer; LFS, Li-Fraumeni syndrome; NA, tumor site not applicable for the syndrome; NR, incidence not reported; PHTS, PTEN hamartoma tumor syndrome; PJS, Peutz-Jeghers syndrome; SI, small intestine.

^a^Percentage of women affected.

### Cumulative Risk Over the Life Course

Information on lifetime cancer risk and relative risk (compared with general population) for some of the RTRS is scarce (**Table 3**). No information was available for familial GIST. In the case of BHD and HLRCC, a lifetime risk of renal cancer was estimated at 20%.^70,75^

**Table 3.** Cancer Cumulative Risk (Range), by Organ and Rare Tumor Risk Syndrome

**RTRS**	**Cancer Risk**
BHD^70^	Renal CA: 20% at age 70
FM^39^	Melanoma: 45%-52% at age 80
HDGC^18,24,73,94^	Gastric CA: 37%-70% (men) and 25%-56% (women) by age 80 Breast CA: 42%-55% by age 80
HLRCC^75^	Renal CA: 21% lifetime risk
LFS^5,25,62^	Any cancer: 100% by age 70. SMN: 50% at 10 years after the 1st cancer diagnosis Breast CA: 56% by age 60 Soft%-tissue sarcoma: 15% (women), 20% (men) by age 60 Brain (men): 20% lifetime ACC: 0.7% at age 2 and 2.21% at age 5
PJS^29,47,53,80,81,83,84,87,95^	Any CA: 43%-76% at age 60 GI CA: 35%-60% at age 60 Breast: 23%-31% at age 60 Gynecological CA: 40% at age 60 Pancreas: 11%-18% at age 60
PHTS^60,61,88–90^	Any CA: 68%-87% (women) and 16%-56% (men) at age 60 Breast CA: 54%-67% at age 60 CRC: 9%-17% lifetime Endometrium: 21%-22% at age 60 Melanoma: 5%-6% lifetime Renal CA: 2%-8% at age 60 Thyroid: 6%-25% at age 60

Abbreviations: ACC, adrenocortical carcinoma; BHD, Birt-Hogg-Dubé syndrome; CA, cancer; CRC, colorectal cancer; FM, familial melanoma; GI, gastrointestinal; GIST, gastrointestinal stromal tumor; HDGC, hereditary diffuse gastric cancer; HLRCC, hereditary leiomyomatosis and renal cell cancer; LFS, Li-Fraumeni syndrome; PHTS, PTEN hamartoma tumor syndrome; PJS, Peutz-Jeghers syndrome; SMN, second malignant neoplasm.

Other RTRS have extensive published data regarding cancer risk in general, as well as the risk of specific types of cancer. For PHTS, 7 of 12 studies reported lifetime cancer risk; for PJS, 9 of 16 studies reported lifetime risk of developing cancer.

Breast cancer risk was one of the most extensively documented in the literature. Patients with HDGC had a mean of 47% breast cancer risk by the age 80,^24,73,94^ with a relatively low risk until 30 to 39 years old but increasing each decade thereafter, corresponding to a 6-fold relative risk compared with the general population. For LFS patients, the risk of breast cancer was 56% at 60 years old, more than 30 times the risk of the general population^62^ and for individuals affected by PJS, it was 11% at 40 years old and 27% at 60.^83,84^ Mean risk of breast cancer for PHTS patients was 4%, 20%, 54%, 60%, and 68% at 30, 40, 50, 60, and 70 years, respectively,^60,61,88^ and similar to LFS patients, almost 30 times higher than in the general population. Ngeow et al^57^ and Hendrix et al^58^ reported 10-year cumulative risks of a second malignant neoplasm after a first breast cancer of 29% and 45.6%, respectively, among women with PHTS. Hendrix et al^58^ further reported a 10-year risk of a PHTS-related second primary cancer of 45.7% in women and 19.8% in men.

Lifetime risk of having any type of cancer can be as high as 100% for LFS and can be 1000 times the general population risk for some types of cancer, such as adrenocortical carcinoma in childhood.^62^

Some of these syndromes were characterized by the occurrence of more than one primary cancer over the course of a lifetime. For instance, in patients with LFS syndrome, the risk of developing a second malignant neoplasm was 50% within 10 years of the initial cancer diagnosis.^46^ Regarding FM, cumulative incidence of a second melanoma was as high as 23%, 5 years after first diagnosis.^38^

PHTS is the RTRS that showed the greatest difference in cancer risk between the sexes, as women have a higher likelihood of developing cancer and tend to be diagnosed at a younger age: Bubien et al^61^ describe the risk of any cancer is 81% for women and 48% for men at age 50 but 85% at age 70 for both sexes.

### Surveillance

Overall, the information available in the literature regarding clinical surveillance is limited (**Table 4**). Out of 80 studies, 35 (43.7%) described a follow-up protocol, 30 (37.5%) reported the outcomes, and only 9 (11.2%) compared the outcomes between participants on a surveillance protocol and those adopting an expectant approach. Moreover, follow-up within some surveillance studies was relatively limited; for example, Castillo-Iturra et al^96^ reported that, on average, each patient underwent only one surveillance endoscopy.

**Table 4.** Number of Studies Containing Data on Clinical Surveillance per Syndrome

**Syndrome (No. of Studies)**	**Surveillance Protocol**	**Surveillance Results**	**Surveillance vs Expectant Management**
BHD (11)	5	1	No data
Familial GIST (1)	No data	No data	No data
FM (9)	5	4	2
HDGC (13)	9	9	1
HLRCC (7)	1	1	No data
LFS (11)	6	6	4
PJS (16)	5	5	1
PHTS (12)	4	4	1
Total	35/80	30/80	9/80

Abbreviations: BHD, Birt-Hogg-Dubé syndrome; FM, familial melanoma; GIST, gastrointestinal stromal tumor; HDGC, hereditary diffuse gastric cancer; HLRCC, hereditary leiomyomatosis and renal cell cancer; LFS, Li-Fraumeni syndrome; PHTS, PTEN hamartoma tumor syndrome; PJS, Peutz-Jeghers syndrome.

Five studies on BHD presented clinical surveillance protocols used in their study populations, with limited detail.^17,66,67,69,97^ Although the only article on familial GIST does not describe the application of any type of clinical surveillance in its population, a proposed protocol is detailed.

FM primarily affects the skin and pancreas; therefore, clinical surveillance focuses on monitoring these two organs. The implementation of surveillance protocols has been associated with the diagnosis of melanoma and pancreatic cancer at earlier stages, making them more likely to be resectable.^64,71^ However, no data on overall survival were available.

For HDGC, there is a strong consensus across studies regarding surveillance protocols. While only one study specified the recommended age to initiate clinical surveillance, and none indicated when it should be discontinued, 9 of 13 studies provided structured protocols. Notably, 48% of patients (mean age, 48; range, 37-64 years) had SRCC detected during surveillance.^48–51,74^ Additionally, a mean of 88% of patients with negative endoscopic results had SRCC identified in risk reducing total gastrectomy (RRTG).^48,74^ Consistent with these findings, Gallanis et al^55^ reported a 49% false-negative rate for the endoscopic detection of SRC lesions.

In the studies by Lee^51^ and Lim,^49^ 28.5% of patients who had positive findings on endoscopy but refused gastrectomy developed macroscopic lesions within 9 to 36 months.

Data on clinical surveillance for HLRCC are scarce, with only 1 of 7 studies describing a screening protocol, which consists of annual renal MRI starting at age 10. In that study by Forde,^75^ 3 patients undergoing active surveillance had renal cell carcinomas detected through screening, all at an early stage. This highlights the potential benefit of systematic screening in identifying tumors at a more treatable stage.

For LFS, 6 of 11 studies reported surveillance protocols. Despite its complexity, given the number of affected organs and the broad scope of surveillance, the studies show a consensus on what and how often clinical surveillance should be performed. However, surveillance outcomes data remain limited. In general, tumors were diagnosed at an asymptomatic stage and tended to be smaller and at an earlier clinical stage. For instance, in the study by Custodio et al,^25^ none of the patients undergoing surveillance for adrenocortical relapsed, and all remained disease-free 31 to 48 months after diagnosis. In contrast, in the nonsurveillance group, tumor size was significantly larger, 25% of patients relapsed, 12% died, and a significant proportion had advanced disease.

Regarding PJS, clinical surveillance begins at birth, but invasive procedures, particularly for the digestive system, are only introduced after the age of 10.^47,52,86^ The reported outcomes varied significantly across studies. While Salloch^47^ found that over 80% of cancers diagnosed during surveillance were low-grade and potentially curable, Korsse^85^ reported that none of the pancreatic tumors (0/7) were detected through surveillance. Similarly, Resta^83^ noted that nearly half of the patients diagnosed with cancer (pancreatic and cervical) during surveillance ultimately died from the disease. It is important to highlight that these studies included a relatively low number of cancer cases (mean, 17; range, 7-42).

Although PHTS is a complex syndrome, only 33% of the studies provided information on clinical surveillance applied to their cohorts, specifically for the detection of uterine, colorectal, thyroid, and breast cancer. In those cases, surveillance led to the diagnosis of breast cancer at an earlier stage, with tumors being less likely to be node-positive.^77^ For detailed information on surveillance protocols, refer to **Appendix, Section 5**.

### Postcancer Survival/Life Expectancy

Survival following a cancer diagnosis is one of the most underreported parameters, with only 23 of 80 studies reporting any data.

In the case of BHD, 4 of 11 studies reported a mean mortality of 16% due to renal cell cancer.^66,67,70,97^ In the case of HLRCC, 4 studies reported a mean survival of 25 months following the diagnosis of metastatic renal cancer.^27,33,35,75^

Patients with FM had a poor prognosis if they were affected by pancreatic cancer, with 70% mortality and a mean postdiagnosis survival of 15.2 months.^71^ In the case of familial GIST, Bachet et al^31^ reported 45% mortality in their cohort.

With only 30% of the studies informing prognosis after cancer diagnosis, data on HDGC prognosis is very limited. While Mi et al^48^ and Gallanis et al^55^ reported 0 deaths in their cohorts (n = 85, 54 CDH1+ and n = 188, respectively), Asif^74^ detailed that 2% died of metastatic breast cancer and Devezas^50^ described that all index cases with advanced diffuse gastric cancer died within a few months.

LFS, despite being one of the RTRS with higher number of possible cancers, only had 2 studies reporting prognosis after cancer diagnosis. De Andrade^62^ showed that by 20 years after first cancer diagnosis, the risk of death before a second cancer is 22.2%. On the other hand, Ferreira^77^ detailed that no patients of the pediatric group developed a second malignant neoplasm after an adrenocortical carcinoma and that 11% of those with adrenocortical carcinoma died of it (median follow-up, 81.5 months; range, 3-378 months). More broadly, Finn et al^45^ reported that 25 of 47 patients died, at a mean age of 44 years (IQR, 38-58).

Patients affected by PJS have a poor prognosis if they were diagnosed with advanced or metastatic cancer, with a mean time from diagnosis to death of 11 months^29,86^ or even less (6 months; IQR, 4-17 months) if they have pancreatic cancer.^85^

With respect to postdiagnosis survival, only one study on PHTS reports a 5-year survival of 100% following total thyroidectomy for minimally invasive follicular carcinoma^92^; however, this observation is based on a single patient.

A significant lack of information on life expectancy in RTRS was observed in the literature, with only 11 of 80 studies reporting on this parameter. Of these, 10 focused on PJS,^28–30,52,53,83–86,95^ although they did not report overall life expectancy but rather the mean age at cancer-related death (range, 40-59 years). For PHTS, one study reported that 69% of deaths were cancer-related, with a median age at death of 36 years (range, 28-71 years).^5^ In another cohort of 513 patients,^44^ 61 deaths occurred at a median age of 55 years (IQR 45-63), of which 27 were cancer-specific.

## DISCUSSION

### Summary of Evidence and Discussion

After a comprehensive and rigorous systematic search, 80 relevant studies were identified and summarized in the present review. In line with the literature on other hereditary syndromes, every study included here was conducted in middle- or high-income countries. More than half of the studies (n = 41; 51.2%) were published after 2018, which relates to technological developments in diagnostic methods and their widespread availability. Notably, in 2005, next-generation sequencing was developed, enabling scientists to expand their understanding of genomics. In 2018, the “100K Genomes” project was launched,^98^ sequencing 100 000 genomes from individuals affected by rare genetic diseases or cancer. These milestones highlight the significant scientific advancements achieved in only recent years and underscore the ongoing need for research.

Although the eight syndromes included were described over a decade ago, included studies do not provide detailed and specific incidence of different tumors, showing a wide range in the proportions of affected individuals. One important consideration in the interpretation of findings on rare hereditary cancer syndromes is the potential for ascertainment bias. The wide variation in reported estimates may, in part, be attributed to differences in how patients are selected for studies. For instance, some studies may focus on individuals with a strong family history of cancer or those identified through specialized clinics, which may not be representative of the broader population affected by these syndromes. Additionally, discrepancies in estimation methods, such as the use of retrospective medical records vs prospective cohort studies, can further contribute to these variations. Recognizing the impact of ascertainment bias is crucial for contextualizing findings and comparing results across different studies.

The data obtained indicate that most of these syndromes have their oncological debut between the fourth and sixth decades of life, although individuals affected by certain RTRS (eg, LFS or PHTS) may develop their first tumor during childhood or adolescence. Notably, patients with *PTEN* mutations develop their second malignant neoplasm in their sixth decade of life in the studies evaluated here, a very similar age at which individuals with other RTRS experience their first (and perhaps even only) cancer. The burden faced by people affected by PHTS exemplifies the complexity of RTRS and underscores the importance of continuing research on these diseases and their treatment/management approaches.

There are clear differences in the availability of information in the literature regarding RTRS. While syndromes such as PJS or LFS have been more extensively studied, with established screening strategies, others have not received the same level of attention. Among the eight syndromes, familial GIST has the fewest identified families and the least available information, with only one study included in the present review, making its continued study particularly pressing.

The lack of available information on the effectiveness of implementing screening programs is noteworthy, specifically the differences observed in patient outcomes diagnosed during the asymptomatic stage versus those diagnosed outside a screening program. Only 9 of 80 (11.2%) studies made a comparison of the outcomes between participants on a surveillance protocol and adopting an expectant approach, with LFS being the condition with most data available, with 40% of the studies reporting results.

### Strengths and Limitations

The limitations of this review follow from the limitations of the body of literature that we sought to summarize.

First, most included studies collected data retrospectively, which may introduce biases and limit the robustness of the findings. Additionally, the number of cases reported in several studies is low, with some including only a few cancer cases, which may result in poor estimates of population parameters. Evidence on surveillance was also limited by the relatively short duration of follow-up reported in some studies.

A second key limitation is the geographic distribution of the studies. The majority originate from high-income countries, likely due to greater access to genetic testing required for patient diagnosis. Consequently, these findings may not be generalizable to lower-income settings, where access to genetic services remains limited, or even certain demographics.

Third is the heterogeneity of the study populations, as some studies included asymptomatic carriers, others only patients with a previous cancer diagnosis, and others both groups. Consequently, reported proportions of patients developing cancer may vary substantially depending on the population studied and should therefore be interpreted with caution.

Fourth, and especially relevant to the stated purpose of informing cost-effectiveness models, is that health state utility values—preference-based quality-of-life measures such as those derived from the EQ-5D or SF-6D and used to calculate quality-adjusted life years (QALYs)—were not reported in any study. This represents a critical evidence gap: without utility estimates, modelers cannot derive quality-adjusted outcomes directly from this review and will need to source utility data from other literature or elicitation exercises.

Fifth, this review reports ranges across studies rather than within-study confidence intervals or standard errors, as these were inconsistently reported in the primary literature. Modelers conducting probabilistic sensitivity analyses should consult the original studies for study-level precision estimates, where available.

Sixth, treatment-focused studies were excluded from this review as they predominantly addressed specific cancer subtypes, disease stages, or therapeutic regimens outside the scope of a natural history mapping exercise. However, such studies may report baseline characteristics and pretreatment natural history data, and control arms in comparative studies may contain longitudinal outcome data relevant to disease progression modeling. Some epidemiological parameters may therefore have been missed.

Lastly, study selection was conducted by a single primary reviewer, which may increase the risk of selection error compared with independent dual screening. However, the preliminary study selection and data extraction were subsequently reviewed by both economic evaluation modelers and syndrome-specific clinical experts within the PREVENTABLE consortium, providing an additional level of verification and clinical oversight.

Despite these limitations, this review provides a detailed, comprehensive and up-to-date mapping of the available evidence across eight RTRS. It synthetizes cancer risk and natural history data across syndromes and organizes cancer outcomes by affected organ to facilitate comparison of disease patterns and clearly identify areas where evidence remains limited or inconsistent. The review also highlights key evidence gaps relevant to future empirical research and the development of cost-effectiveness models.

## CONCLUSION

Management of individuals with RTRS remains a significant challenge due to the wide spectrum of associated cancer risks and the need for tailored surveillance strategies. Cost-effectiveness analyses comparing alternative management approaches are therefore important to support patient care and resource allocation.

This review provides modelers with a structured synthesis of several key inputs relevant to economic evaluation, including age at syndrome and cancer diagnosis, cancer-specific and cumulative risks, patterns of multiple primary malignancies, surveillance strategies and outcomes, and available information on prognosis and mortality. However, important parameters remain poorly characterized for several RTRS, particularly long-term surveillance outcomes, life expectancy, and the comparative effectiveness of alternative surveillance strategies. Notably, health state utility values, which are essential for QALY calculation in cost-effectiveness analysis, are absent from the existing literature for these syndromes; this gap should be prioritized in future primary research.

Where robust estimates are unavailable, modelers may need to combine the evidence identified in this review with expert clinical input, registry or real-world data, and appropriate sensitivity or scenario analyses to reflect parameter uncertainty. By mapping both the available evidence and the remaining gaps, this review provides a practical evidence base for future cost-effectiveness models and highlights priorities for further empirical research.

### Declarations

The authors declare no conflicts of interest.

### Ethical Statement

This study is a review of previously published literature and did not involve human participants or the collection of primary data. Therefore, ethical approval was not required.

### PREVENTABLE Consortium

Ricardo Amorim, Stefan Aretz, Ana Azevedo, Judith Balmaña, Stéphanie Bart-Desurmont, Birte Blindheim Lundhaug, Paula Boaventura, Gaëlle Bougeard, Maud Branchaud, João Paulo Brás, Adriana Costal, Carolina Di Fabrizio, Núria Dueñas, Laura Duran-Lozano, Patricia Faure, Isabel Fernandes, Daniel Ferreira, Diana Ferreira, Marbélia Fernandes, João Fonseca, Judite Gonçalves, Cécile Guillemet, Nicoline Hoogerbrugge, Claude Houdayer, Robert Hüneburg, Edwige Kasper, Silvana Lobo, Adrià López-Fernández, Margaux Clément Le Choismier, Luzia, Ana Machado, Marta M. Marques, Céu Mateus, Ana Rita Matos, António Moreira, Maiara Moreto, Amalia Nicole Nanciu, Carla Oliveira, Rebecca - Evelyn Papp, Nathalie Parodi, Ana Maria Pedro, Bárbara Peleteiro, Sara Pereira, Mia Quante, Mariette Renaux-Petel, Ana Maria Rodrigues, Marion Rolain, Celina São José, Janneke H.M. Schuurs-Hoeijmakers, Zillur Rahman Shabuz, Paula Soares, Liliana Sousa, Rui Sousa, Alexander Sun Zhang, Jean-Christophe Théry, Andrew Titman, Katrin van Beekum, Martine P.A. van Koolwijk, Katja C.J. Verbeek, Thomas Vermeulin, Hildegunn Høberg Vetti, João Vinagre.

## Supporting information

Online Supplementary Material

## Data Availability

All data generated or analyzed during this study are included in this published article and its supplementary material.
