# Supplementary material for: Natural History of Rare Tumor Risk Syndromes: A Scoping Review to Inform Cost-Effectiveness Models
Source: J Health Econ Outcomes Res. 2026 Oct 12;13(2):135–47. doi: 10.36469/001c.167564 (PMC13666261; doi:10.36469/001c.167564)
Supplement: Supplementary file 1 [file jheor_2026_13_2_167564_367878.pdf]

Journal of Health Economics and Outcomes Research

Online Supplementary Material

Natural History of Rare Tumor Risk Syndromes: A Scoping Review to Inform Cost-Effectiveness Models. *JHEOR*. 2026;13(2):??-??. doi:10.36469/jheor.2026.167564

Section 1: Search Strategy **1**

Section 2: Preferred Reporting Items for Systematic Reviews and Meta-Analyses Extension for Scoping Reviews (PRISMA-ScR) Checklist **3**

Section 3: Results **6**

Section 4: Summary Tables of the Included Studies' Characteristics, by Syndrome **14**

Section 5: Surveillance **21**

This supplementary material has been provided by the authors to give readers additional information about their work.

This is an open-access article distributed under the terms of the Creative Commons Attribution 4.0 International License (CCBY-4.0). View this license's legal deed at http://creativecommons.org/licenses/by/4.0 and legal code at http://creativecommons.org/licenses/by/4.0/legalcode for more information.

Section 1. Search Strategy

1.1. Search Terms

Birt-Hogg-Dubé Syndrome (BHD):
“Birt-Hogg-Dubé” OR “Hornstein-Knickenberg” OR “Folliculin” OR “FCNL”
Last search performed April 6, 2026

Familial Melanoma (FM)
"Familial melanoma" OR "Familial malignant melanoma" OR "Familial atypical multiple mole melanoma" OR "FAMMM”
Last search performed April 6, 2026

Familial GastroIntestinal Stromal Tumour (GIST)
(“KIT” OR “PDGFRA”) AND (“GIST” OR "Gastrointestinal stromal tumour" OR "gastrointestinal stromal tumor" OR "familial GIST")
Last search performed April 6, 2026

Hereditary Diffuse Gastric Cancer (HDGC)
"Hereditary diffuse gastric cancer" OR "Hereditary lobular breast cancer" OR "HDGC" OR "Germline CDH1"
Last search performed April 6, 2026

Hereditary Leiomyomatosis and Renal Cell Cancer (HLRCC)
"Hereditary Leiomyomatosis and Renal Cell Cancer" OR "Hereditary leiomyomatosis" OR "FH tumour predisposition syndrome" OR "HLRCC"
Last search performed April 6, 2026

Li Fraumeni Syndrome (LFS)
"Li Fraumeni" OR "Germline TP53"
Last search performed April 6, 2026

Peutz-Jeghers Syndrome (PJS)
"Peutz-Jeghers" OR "STK11" OR "LKB1"
Last search performed April 6, 2026

PTEN Hamartoma Tumour Syndrome (PHTS)
"PTEN Hamartoma Tumour Syndrome" OR “PHTS” OR "Hamartoma Tumour Syndrome" OR "Germline PTEN"
Last search performed April 6, 2026

1.2. Inclusion and Exclusion Criteria

Table 1: Inclusion and Exclusion Criteria

Inclusion	Exclusion
Patients diagnosed with the included RTRS (both symptomatic and asymptomatic carriers)	Special populations (pregnant women, children)
Studies reporting data on natural history (age of diagnosis, cancer risk)	Studies focused on treatments (surgical techniques, drugs, radiotherapy), diagnostic techniques or genotype-phenotype association
Cancer incidence, age of onset, post diagnosis survival, surveillance effectiveness	No clinical or epidemiological outcomes reported
Original research articles including cohort, case-control, or cross-sectional studies	Not research papers, single case/family reports, reviews and meta analysis
Written in English, Spanish or Portuguese	
Published between 2010 and 2026	

Section 2: Preferred Reporting Items for Systematic Reviews and Meta-Analyses Extension for Scoping Reviews (PRISMA-ScR) Checklist

Table 2: PRISMA for Scoping Reviews Checklist

Section	Item	PRISMA-ScR CHECKLIST ITEM	REPORTED ON PAGE NO.
		**TITLE**	
**Title**	1	Identify the report as a scoping review.	1
		**ABSTRACT**	
**Structured summary**	2	Provide a structured summary that includes (as applicable): background, objectives, eligibility criteria, sources of evidence, charting methods, results, and conclusions that relate to the review questions and objectives.	2
		**INTRODUCTION**	
**Rationale**	3	Describe the rationale for the review in the context of what is already known. Explain why the review questions/objectives lend themselves to a scoping review approach.	4
**Objectives**	4	Provide an explicit statement of the questions and objectives being addressed with reference to their key elements or other relevant key elements used to conceptualize the review questions and/or objectives.	5
		**METHODS**	
**Protocol and registration**	5	Indicate whether a review protocol exists; state if and where it can be accessed; and if available, provide registration information, including the registration number.	6
**Eligibility criteria**	6	Specify characteristics of the sources of evidence used as eligibility criteria and provide a rationale.	6
**Information sources**	7	Describe all information sources in the search, as well as the date the most recent search was executed.	6
**Search**	8	Present the full electronic search strategy for at least 1 database, including any limits used, such that it could be repeated.	Appendix
**Selection of sources of evidence**	9	State the process for selecting sources of evidence included in the scoping review.	7

Section	Item	PRISMA-ScR CHECKLIST ITEM	REPORTED ON PAGE NO.
**Data charting process**	10	Describe the methods of charting data from the included sources of evidence and any processes for obtaining and confirming data from investigators.	7
**Data items**	11	List and define all variables for which data were sought and any assumptions and simplifications made.	7
**Critical appraisal of individual sources of evidence**	12	If done, provide a rationale for conducting a critical appraisal of included sources of evidence; describe the methods used and how this information was used in any data synthesis (if appropriate).	NA
**Synthesis of results**	13	Describe the methods of handling and summarizing the data that were charted.	8
**RESULTS**			
**Selection of sources of evidence**	14	Give numbers of sources of evidence screened, assessed for eligibility, and included in the review, with reasons for exclusions at each stage, ideally using a flow diagram.	9, Appendix
**Characteristics of sources of evidence**	15	For each source of evidence, present characteristics for which data were charted and provide the citations.	9
**Critical appraisal within sources of evidence**	16	If done, present data on critical appraisal of included sources of evidence.	NA
**Results of individual sources of evidence**	17	For each included source of evidence, present the relevant data that were charted that relate to the review questions and objectives.	10, Appendix
**Synthesis of results**	18	Summarize and/or present the charting results	10, Appendix
**DISCUSSION**			
**Summary of evidence**	19	Summarize the main results (including an overview of concepts, themes, and types of evidence available), link to the review questions and objectives, and consider the relevance to key groups.	25
**Limitations**	20	Discuss the limitations of the scoping review process.	27
**Conclusions**	21	Provide a general interpretation of the results with respect to the review questions and objectives, as well as potential implications and/or next steps.	28

Section	Item	PRISMA-ScR CHECKLIST ITEM	REPORTED ON PAGE NO.
**FUNDING**			
**Funding**	22	Describe sources of funding for the included sources of evidence, as well as sources of funding for the scoping review. Describe the role of the funders of the scoping review.	30

Section 3: Results

3.1- Data Charting Tables

Figure 1: Birt-Hogg-Dubé Syndrome, Data Charting Table

BIRT HOGG DUBE

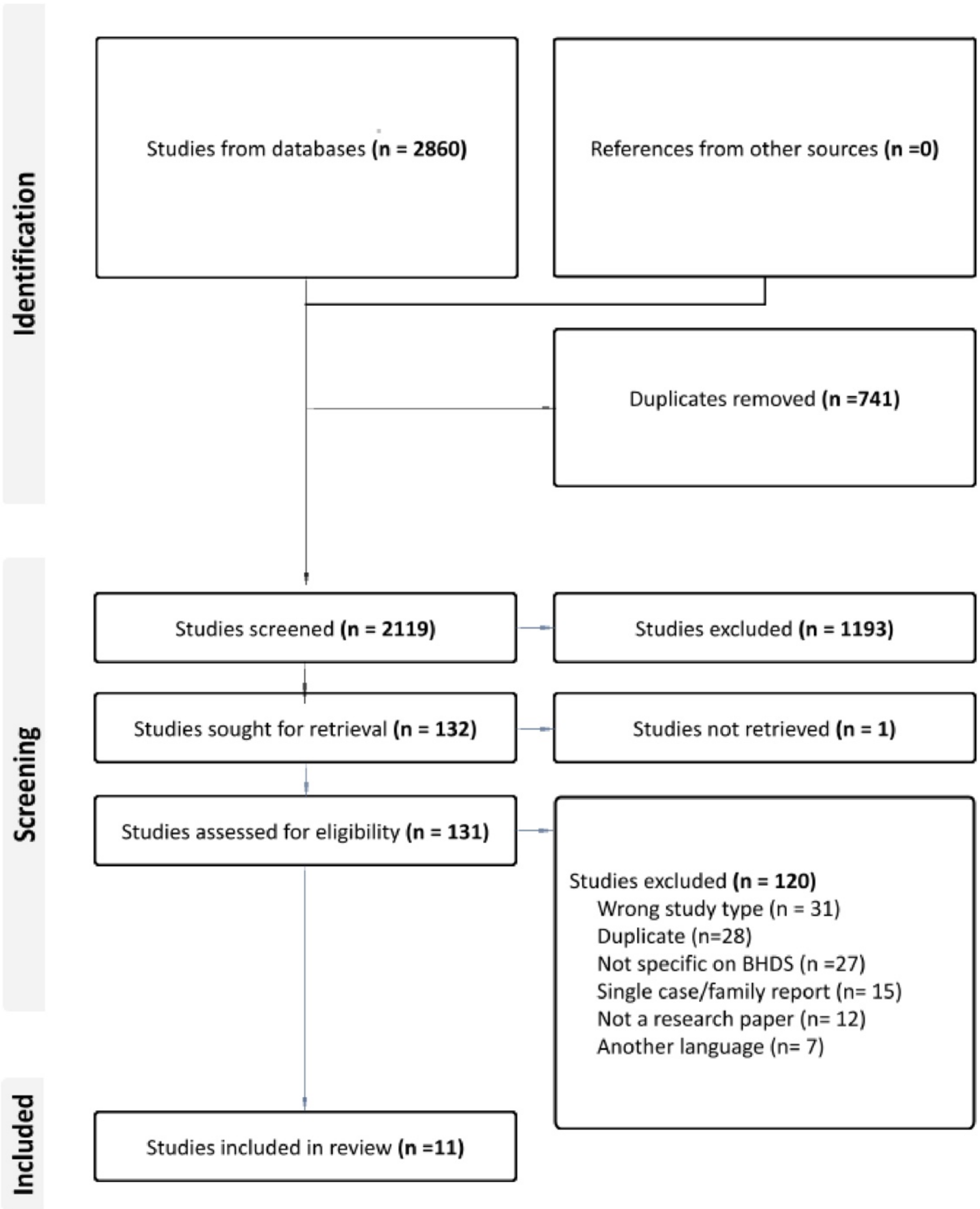

16th August 2026 covidence

Figure 2: Familial Melanoma, Data charting table

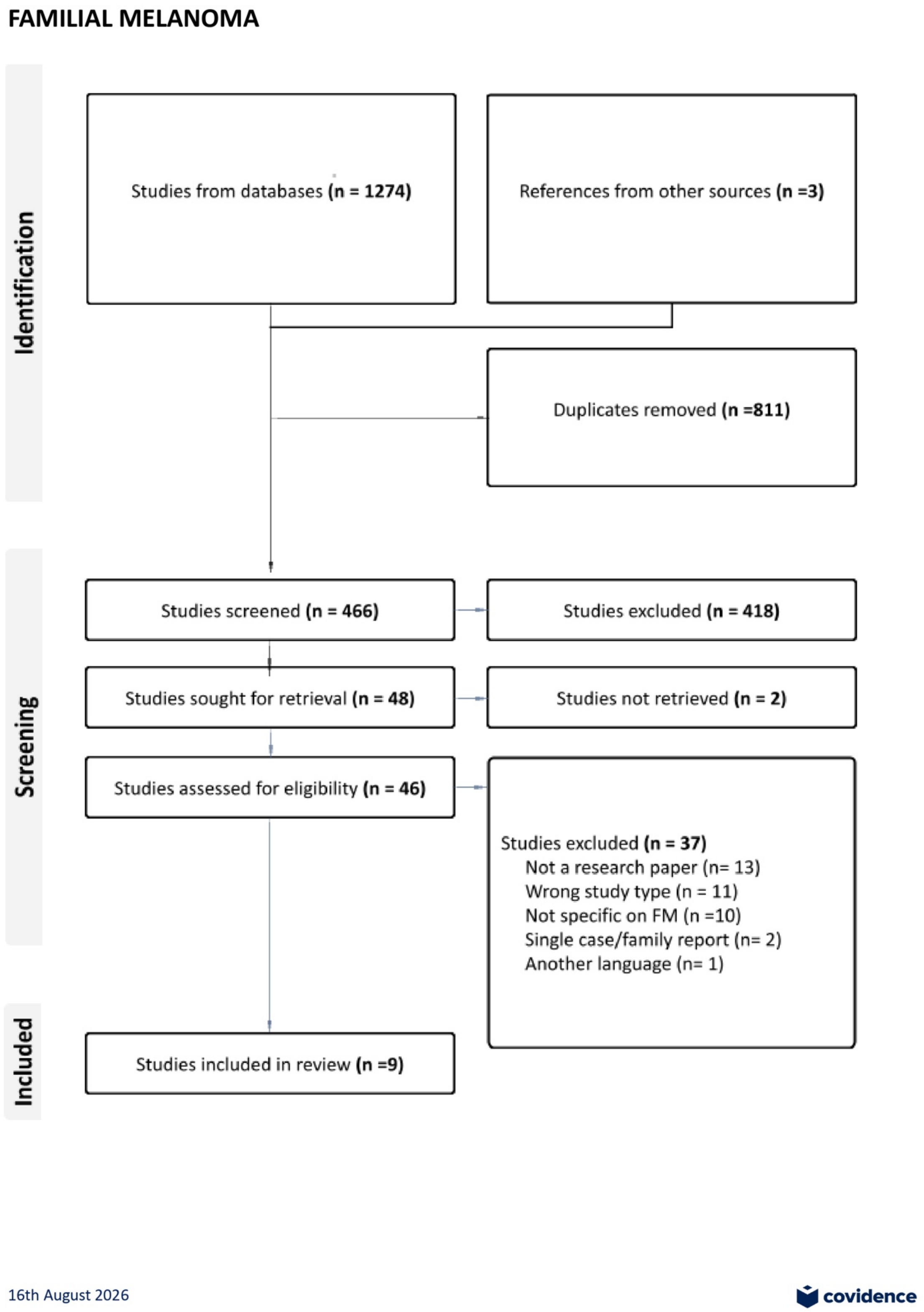

Figure 3: Familial Gastrointestinal Stromal Tumor, Data Charting Table

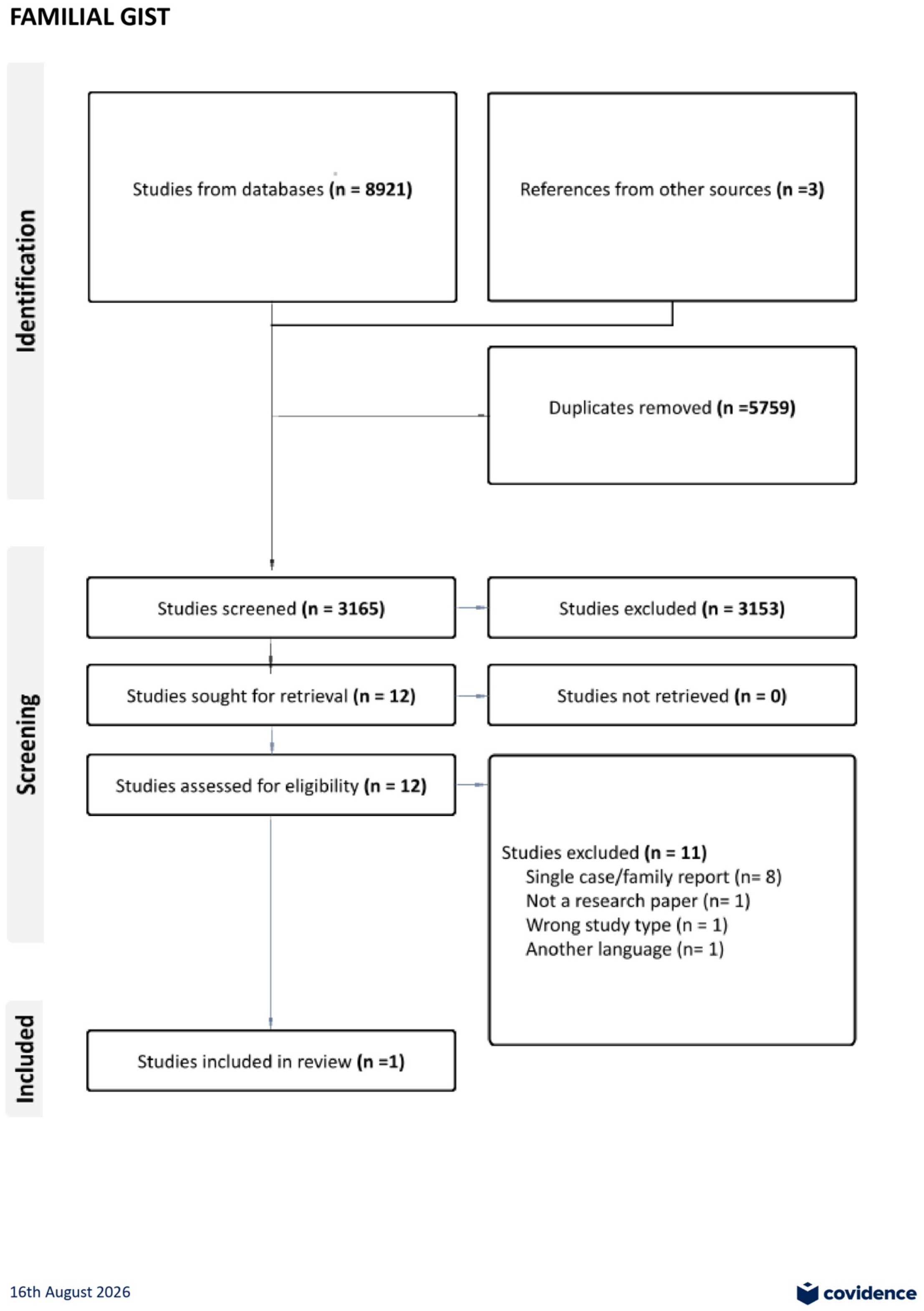

Figure 4: Hereditary Diffuse Gastric Cancer, Data Charting Table

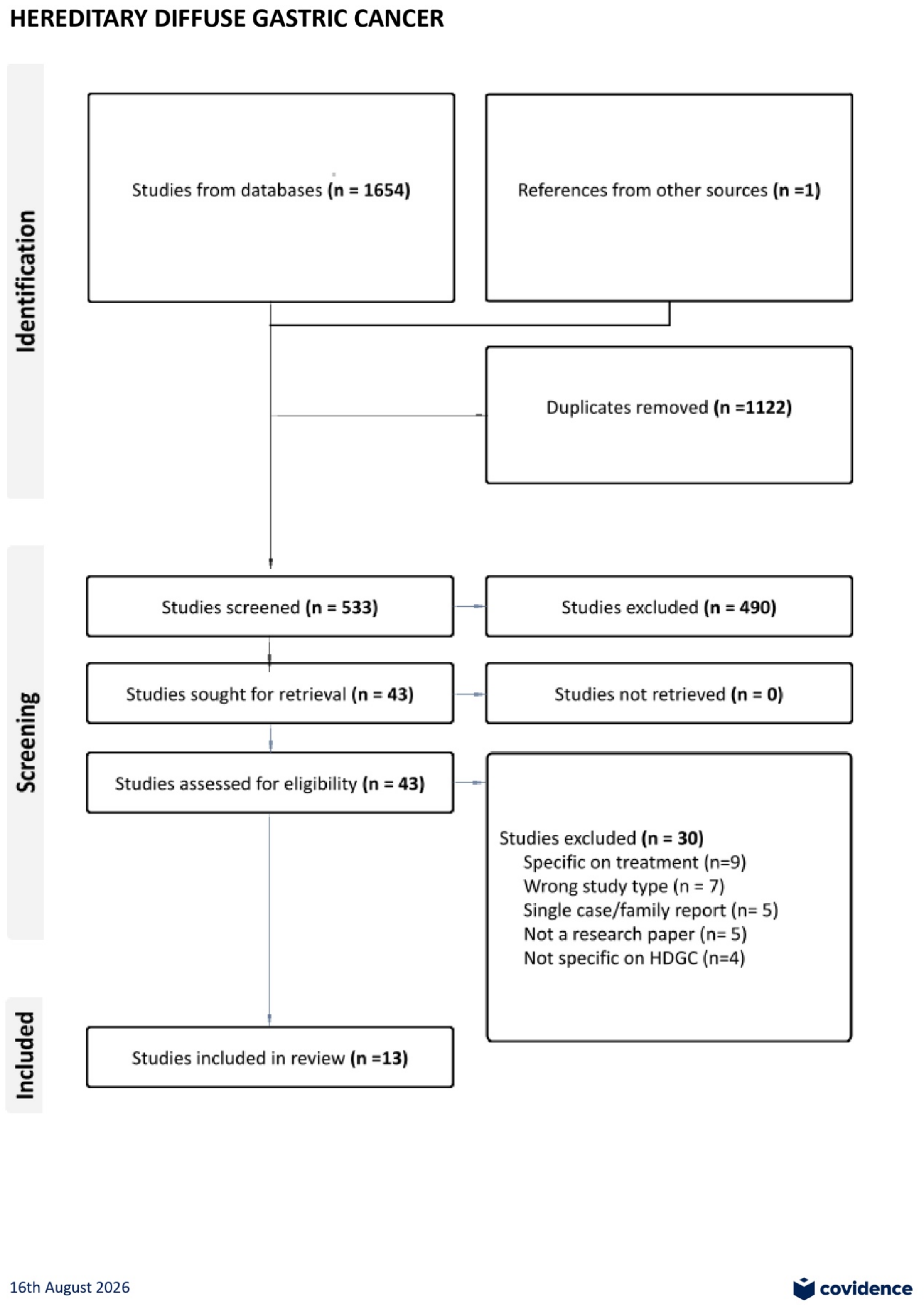

Figure 5: Hereditary Leiomyomatosis and Renal Cell Cancer, Data Charting Table

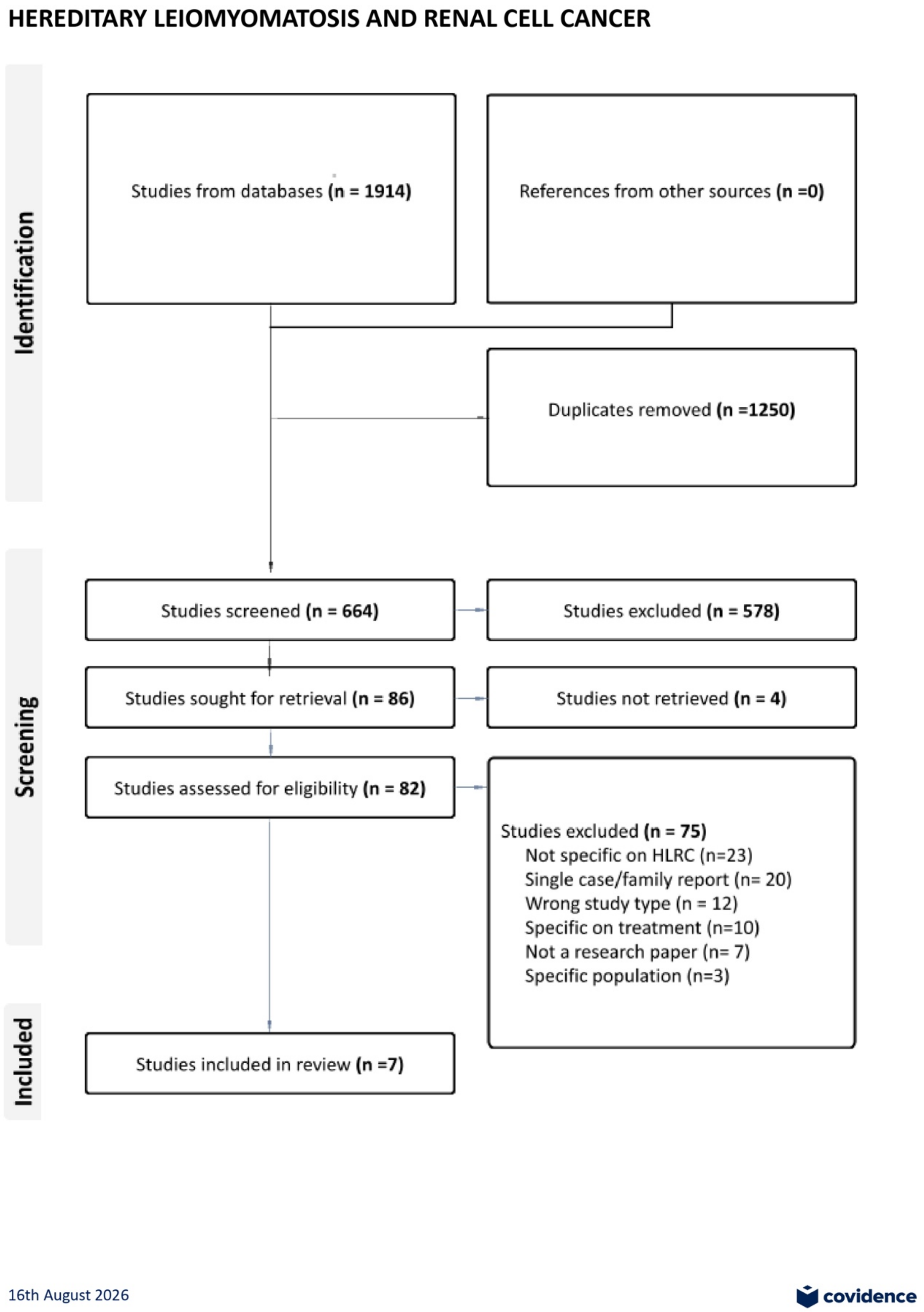

Figure 6: Li-Fraumeni Syndrome, Data Data Charting Table

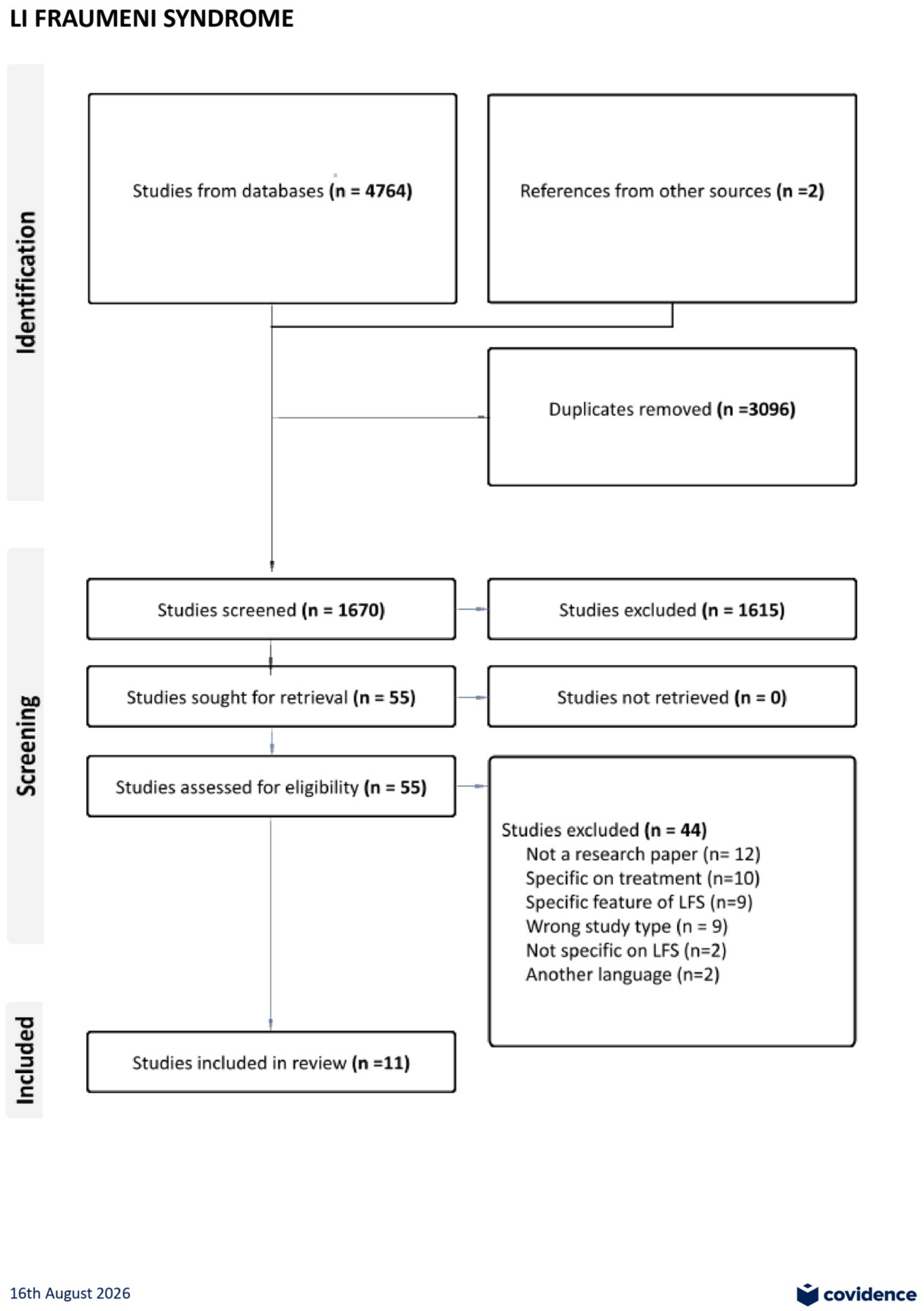

Figure 7: Peutz-Jeghers Syndrome, Data Charting Table

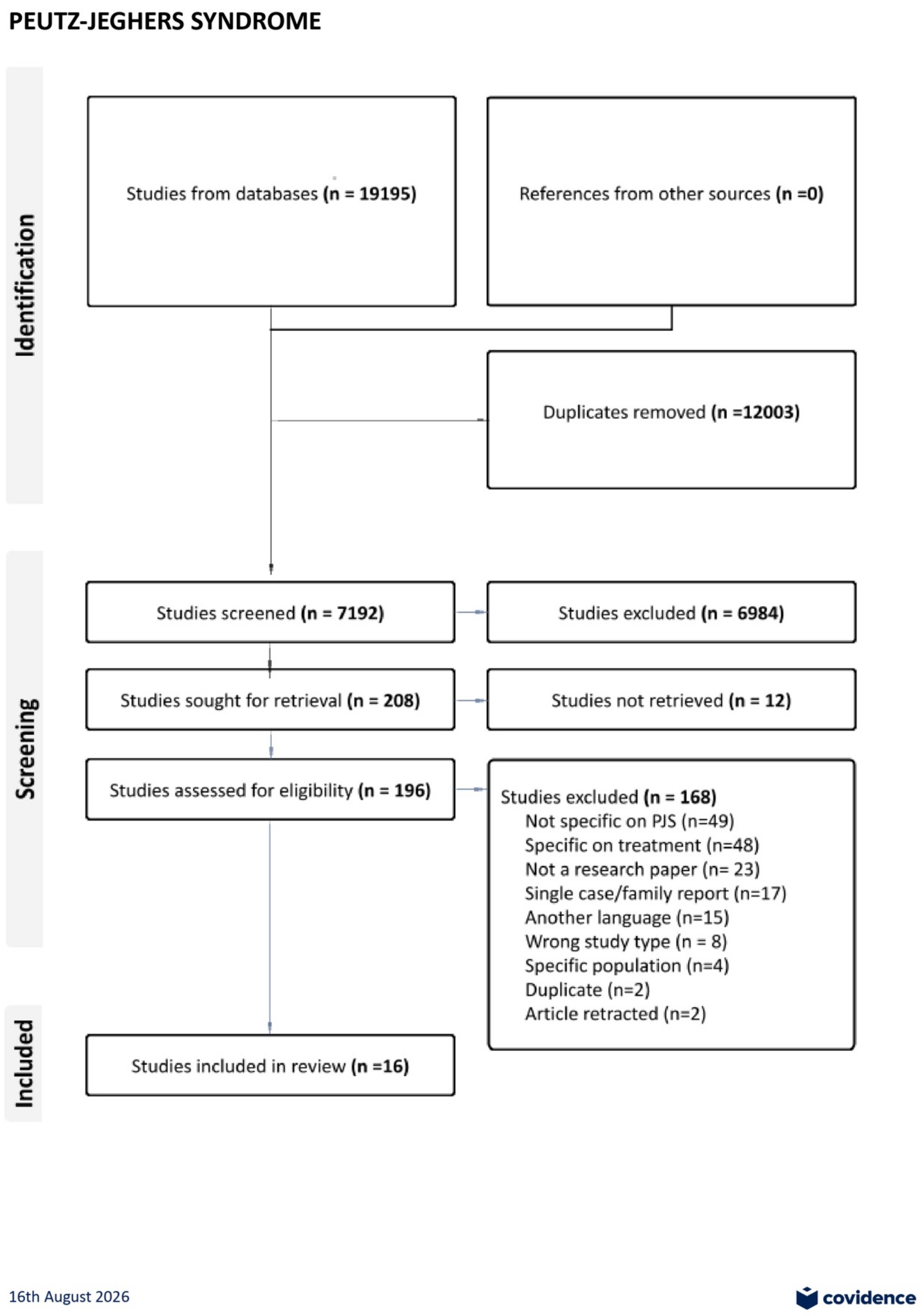

Figure 8: *PTEN* Hamartoma Tumor Syndrome, Data Charting Table

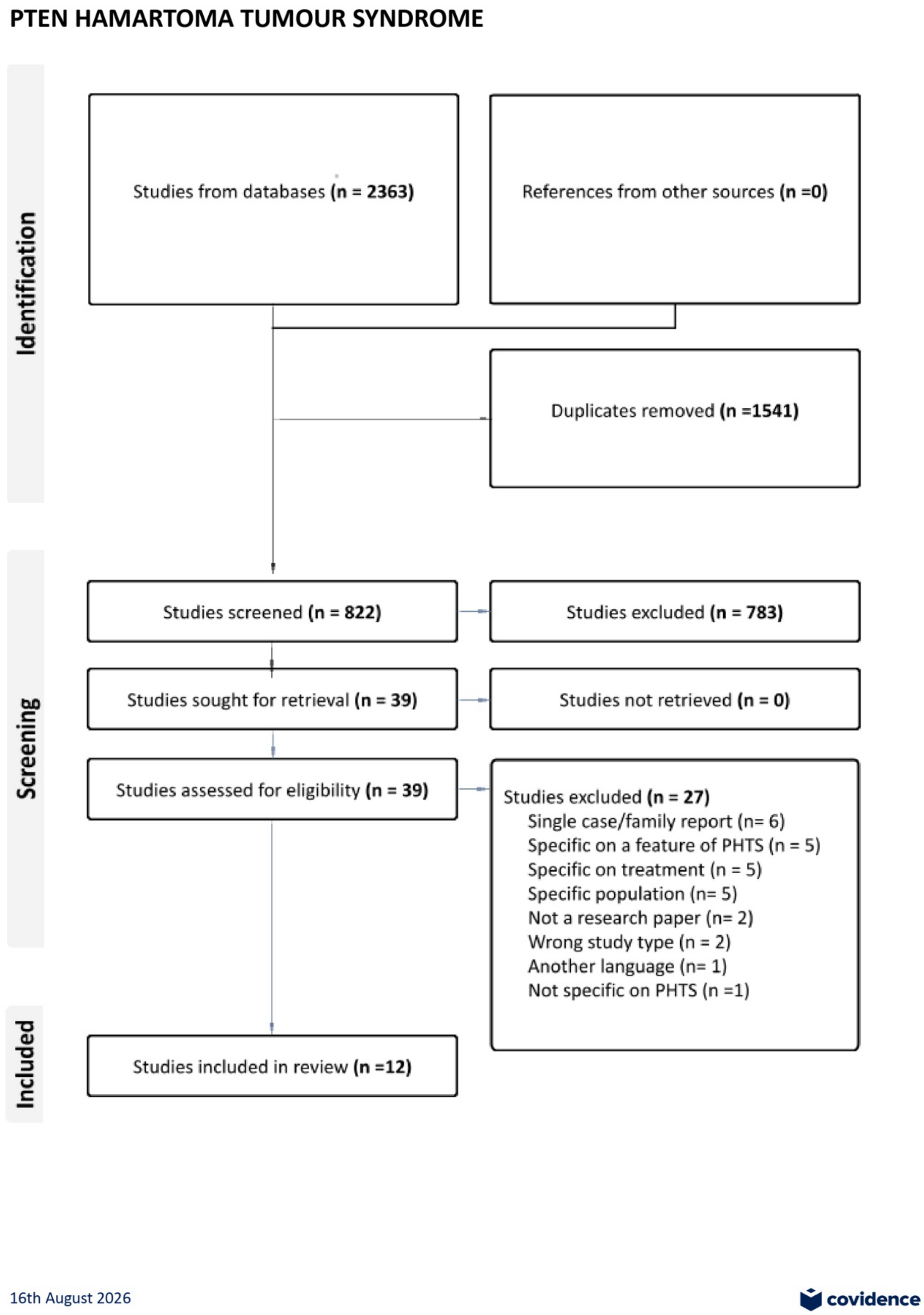

Section 4: Summary Tables of the Included Studies' Characteristics, by Syndrome

Table 3: Characteristics of the Included Studies on Birt-Hogg-Dubé Syndrome

Author	Year	Country	Study Type	No. of Patients	Sex (% Female)	Age (Years)	Symptomatic Patients	Carriers	Follow-up (Years)/Study Duration
Benusiglio[97]	2014	France	RC	33	36.4	NR	Yes	No	M: 9.1
Daccord[69]	2020	Switzerland	RC	96	48	M: 48 (SD 14)	Yes	No	6
Furuya[67]	2016	Japan	RC	312	NR	NR	Yes	Yes	NR
Houweling[70]	2011	Netherlands	RC	115	NR	NR	Yes	Yes	NR
Johannesma[66]	2019	Netherlands	RC	199	NR	M: 50 (r 20-83)	Yes	Yes	M: 4.2y (r1-9)
Lee[16]	2018	South Korea	RC	12	66,70	M: 41.3 ± 12.0	Yes	Yes	Med: 4.3 (r 0.1-12.4)
Liu[99]	2017	China	RC	27	89	Med: 48 (r29-65)	Yes	No	3
Namba[68]	2023	Japan	RC	334	58.7	Med: 44 (r 16–88)	Yes	Yes	No follow up
Park[25]	2022	South Korea	RC	26	57.7	M: 51 (IQR 34-58)	Yes	No	NR
Hu[36]	2024	China	RC	100	63	M: 45.3(r 15-81)	Yes	No	No follow up
Kijlertsuphasri[56]	2024	USA	RC	149	53	M: 56,0 (+-15,2)	Yes	No	3

Abbreviations: IQR, interquartile range; M, mean; Med, median; NR, not reported; r, range; RC, retrospective cohort; SD, standard deviation.

Table 4: Characteristics of the Included Studies on Familial Melanoma

Author	Year	Country	Study Type	No. of Patients	Sex (% Female)	Age (Years)	Symptomatic Patients	Carriers	Follow-up (Years)/Study Duration
Cust[39]	2011	Australia-UK	Case Controls	580	NR	NR	Yes	Yes	NR
Pissa[63]	2022	Sweden	RC	473	51.2%	NR	Yes	Yes	34
Potjer[65]	2015	Netherlands	Case Controls	185	63.2%	(Med) Cases: 55 (r 21-76)<br>Controls: 64 (r 55–88)	Yes	Yes	16
Potjer[40]	2015	Netherlands	PC	150	57.3%	Med: 51 (r 36–72)	Yes	Yes	Med: 3.6 (r 0.1-12)
Potrony[100]	2014	Spain	RC	702	NR	NR	Yes	No	21
Sá[64]	2017	Brazil	PC	124	49.2%	NR	Yes	No	5
Sargen[15]	2021	USA	Case Controls	293	46%	NR	Yes	No	38
van der Rhee[36]	2010	Netherlands	RC	182	56%	NR	Yes	No	Med: 10.4 (r 0-35.7)
Vasen[71]	2011	Netherlands	PC	79	57%	M: 56 (r 39 –72)	No	Yes	Med: 4 (r 0 –10)

Abbreviations: IQR, interquartile range; M, mean; Med, median; NR, not reported; PC, prospective cohort; r, range; RC, retrospective cohort.

Table 5: Characteristics of the Included Studies on Familial Gastrointestinal Stromal Tumor

Author	Year	Country	Study Type	No. of Patients	Sex (% Female)	Age (Years)	Symptomatic Patients	Carriers	Follow-up (Years)/Study Duration
Bachet[30]	2013	France	RC	20	50	M: 36.5 (r 17-46)	Yes	No	NR

Abbreviations: M, mean; NR, not reported; r, range; RC, retrospective cohort.

Table 6: Characteristics of the Included Studies on Hereditary Diffuse Gastric Cancer

Author	Year	Country	Study Type	No. of Patients	Sex (% Female)	Age (Years)	Symptomatic Patients	Carriers	Follow-up (Years)/Study Duration
Asif[74]	2023	USA	PC	270	64	Med: 46.6 (IQR 36.5 – 59.8)	No	Yes	Med: 2.6 (IQR 1.4–3.5)
Devezas[50]	2020	Portugal	RC	34	52.9	M: 40.8 (r 14-83)	No	Yes	13
Gamble[72]	2022	USA	PC	283	70.3	Med: 48 (r 18-81)	Yes	Yes	4
Hansford[94]	2015	Canada	RC	183	NR	NR	Yes	Yes	7
Lee[51]	2023	UK	PC	145	53	Med: 43.3 (IQR 32.6 – 55.9)	Yes	Yes	Med 4.2 (IQR 1.5-6.7)
Lim[49]	2013	UK	PC	29	44.8	Med: 36 (r 19 – 65)	No	Yes	Med 1.7 (IQR 0.9-5.2)
Mi[48]	2017	UK	PC	85	49.4	Med: 38 (IQR 28-50.5)	No	Yes	Med: 1 (IQR 0-3)
Roberts[73]	2019	USA	RC	238	63.4	M: 49.3 (SD 18.1)	Yes	Yes	5
Xicola[24]	2019	USA	PC	113	NR	M: 50 (SD 14)	Yes	Yes	5
Castillo Iturra[96]	2025	Spain	RC	53	45.3	Med: 39 (IQR 25-52)	No	Yes	16
Gallanis[55]	2025	USA	PC	188	70	Med: 31 (r 8-39)	Yes	Yes	6
Mejia Perez[101]	2024	USA	RC	48	68.7	Med (at genetic testing): 45,5 [IQR 33, 57.5].	No	Yes	15
Ryan[18]	2024	USA	RC	883	62	Age range, 17-84	Yes	Yes	21

Abbreviations: IQR, interquartile range; M, mean; Med, median; NR, not reported; PC, prospective cohort; r, range; RC, retrospective cohort.

Table 7: Characteristics of the Included Studies on Hereditary Leiomyomatosis and Renal Cell Cancer

Author	Year	Country	Study Type	No. of Patients	Sex (% Female)	Age (Years)	Symptomatic Patients	Carriers	Follow-up (Years)/Study Duration
Bhola[34]	2018	Canada	RC	48	60.4	M 39.1 (r 9-91)	Yes	No	12
Forde[75]	2020	UK	RC	185	57.8	NR	Yes	Yes	NR
Muller[35]	2017	France	RC	182	63	NR	Yes	Yes	M: 2.7 (r 0.1-11.2)
Sánchez-Heras[27]	2020	Spain	RC	197	57.4	M: 51.1 (SD 13.4)	Yes	Yes	10
Scharnitz[33]	2023	USA	RC	57	71.9	NR	Yes	Yes	13
Seo[42]	2021	South Korea	RC	13	46.1	NR	Yes	No	1.7
Smit[41]	2011	Netherlands	RC	35	NR	NR	Yes	Yes	No follow-up

Abbreviations: M, mean; NR, not reported; PC, prospective cohort; r, range; RC, retrospective cohort; SD, standard deviation.

Table 8: Characteristics of the Included Studies on LFS

Author	Year	Country	Study Type	No. of Patients	Sex (% Female)	Age (Years)	Symptomatic Patients	Carriers	Follow-up (Years)/Study Duration
Bojadzieva[78]	2017	USA	RC	63	71.4	0–20: 22%-21–40: 41% 41–60: 26%->60: 11%	Yes	Yes	Med: 1.3 (IQR 0.5–2)
Bougeard[59]	2015	France	RC	415	61.9	NR	Yes	Yes	20
Custodio[25]	2013	Brazil	RC	461	NR	r 0-15	No	Yes	5
de Andrade[62]	2021	USA	RC	480	62.3	Med: 36.7 (fem), 34.3 (males)	Yes	Yes	9
Ferreira[77]	2019	Brazil	RC	51	67	Med: 3 (r0.8-60)	Yes	No	Med: 3 (r 0.1-31.5)
Mai[5]	2016	USA	RC	286	65	Med: 35 (r 0-91)	Yes	Yes	NR
Mai[46]	2017	USA	PC	116	66.4	Med: 37.6 (r 3-68)	Yes	Yes	4
Saya[79]	2017	UK	RC	88	61	Med: 38	Yes	Yes	4
Villani[32]	2011	Canada, USA	PC	33	42.4	NR	No	Yes	6
Villani[76]	2016	Canada, USA	PC	89	57.3	Med (2 groups): 23.0 and 18.0	Yes	Yes	Med: 2.7
Finn[45]	2025	UK	RC	57	79	(at LFS diagnosis) Med: 38 (IQR 29-48)	Yes	Yes	Med follow up: 6 (IQR 2-11)

Abbreviations: IQR, interquartile range, Med, median; NR, not reported; PC, prospective cohort; r, range; RC, retrospective cohort; SD, standard deviation.

Table 9: Characteristics of the Included Studies on Peutz-Jeghers Syndrome

Author	Year	Country	Study Type	No. of Patients	Sex (% Female)	Age (Years)	Symptomatic Patients	Carriers	Follow-up (Years)/Study Duration
Chen[80]	2017	China	RC	336	46.1	NR	Yes	No	11
Chiang[54]	2018	Taiwan	RC	15	53.3	NR	Yes	Yes	M: 16.5 (r 5-28)
Jelsig[30]	2016	Denmark	RC	43	39.5	Med: 35.5 (r 2–78)	Yes	Yes	NR
Jelsig[29]	2023	Denmark	RC	56	37.5	Med: 34	Yes	No	NR
Jelsig[82]	2023	Denmark	RC	52	42	NR	Yes	No	M: 24.2
Kim[87]	2023	South Korea	RC	96	50	Med: 34.7 (10.5–81.2)	Yes	No	Med: 6.2 (r 0–22.5)
Korsse[85]	2013	Netherlands	PC	144	51	Med: 37 (IQR 21-52)	Yes	Yes	16
Resta[83]	2013	Italy	RC	119	51.3	NR	Yes	Yes	12
Salloch[47]	2010	Germany	PC	31	65	Med:40 (r 10-66)	Yes	No	7
Tchekmedyian[28]	2013	Uruguay	RC	25	40	r 12-65	Yes	No	9
Van Lier[52]	2011	Netherlands	PC	133	52	Med: 34 (r 4-75)	Yes	Yes	14
Van Lier[95]	2010	Netherlands	PC	110	54	M: 35 (r 4–75)	Yes	Yes	14
Wang[53]	2011	China	RC	25	81.8	M: 31.7 (r 19-67)	Yes	No	12.7
Wang[84]	2022	China	PC	412	43.4	Med: 30 (r 1-73)	Yes	Yes	15
Xu[81]	2023	China	RC	566	44.7	Med: 15 (r 0.5-60)	Yes	No	28
You[86]	2010	USA	RC	54	50	NR	Yes	No	Med: 7 (IQR: 2–20)

Abbreviations: IQR, interquartile range, Med, median; NR, not reported; PC, prospective cohort; r, range; RC, retrospective cohort.

Table 10: Characteristics of the Included Studies on *PTEN* Hamartoma Tumor Syndrome

Author	Year	Country	Study Type	No. of Patients	Sex (% Female)	Age (Years)	Symptomatic Patients	Carriers	Follow-up (Years)/Study Duration
Bubien[61]	2013	France	RC	146	47.9	Med: 36 (r 0.5–76)	Yes	Yes	11
Drissen[93]	2022	Netherlands	RC	84	63	Med: 37 (IQR 23–43)	Yes	Yes	Med 3 (IQR 1-4)
Drissen[92]	2023	Netherlands	RC	37	65	Med: 47 (IQR 43–55)	Yes	Yes	Med 3 years (IQR 2-5)
Hendricks[88]	2023	Netherlands (multicenter)	RC	455	61.8	Med: 44 (IQR 33-55)	Yes	Yes	Med: 6 (IQR 3-10)
Hoxhaj[91]	2022	Netherlands	RC	65	100	Med: 38 (r 24–70)	Yes	Yes	Med: 4 (r 1–15)
Kwinten[102]	2023	Netherlands	RC	25	100	Med: 39 (r 31–60)	Yes	Yes	Med: 3.2 (r 0.5–8)
Ngeow[57]	2014	USA (Multicenter)	PC	114	79.8	Med: 40.5 (r 9-69)	Yes	No	7
Nieuwenhuis[60]	2014	Netherlands (multicenter)	RC	180	55	M: 32 (r 1–73)	Yes	Yes	NR
Tan[89]	2012	USA (Multicenter)	PC	368	56	Med: 39 (r 0.4–83)	Yes	Yes	10
Yehia[90]	2023	USA (Multicenter)	PC	701	59	Med: 38 (IQR 12-52)	Yes	Yes	Med: 4 (IQR 2-8)
Hendricks[58]	2025	Netherlands	RC	279	80	At last follow up. Med: females: 51 (IQR 40-60), males: 56 (IQR 46-64)	Yes	No	Med follow-up after 1st cancer: 5 (Females IQR = 1-11, males: 1-12)
Hendricks[44]	2025	Netherlands	RC	513	65	At last follow up: Med: Females 50 (IQR 41–59) Males: 58 (IQR 50–64)	Yes	Yes	4

Abbreviations: IQR, interquartile range, M, mean; Med, median; NR, not reported; PC, prospective cohort; r, range; RC, retrospective cohort.

Section 5: Surveillance

Table 11: Surveillance Protocols Applied in the Included Studies

Syndrome	Organ	Method	Start	Frequency	References
**BHD**	Lung	HRCT	Age 20 or BHD diagnosis	Annual	16, 65, 66, 68, 95
	Kidney	Abdominal CT (100 sensitivity) or US (50 sensitivity)			
**Familial GIST** (Recommended protocol, not applied in the article)	GIT	Abdominal CT or MRI	Age 18	-If no symptomatic tumor or <2 cm: every 2–3 years<br>-If symptomatic tumor or >2 cm: at 6 months, then annually.	31
**FM**	Skin	Total skin examination + dermoscopy	Proband or relatives with melanoma: Since diagnosis	Every 6 months	15, 39, 62,63, 70
			Asymptomatic relatives: Since childhood	Annually	
	Pancreas	MRI/MRCP	Age 45	Annual. If clear neoplastic lesions: surgery. If doubt: repeat MRI/MRCP in 2-4 months.	
**HDGC**	Stomach	Endoscopy (with random and targeted biopsies)	Since HDGC diagnosis	<u>CDH1+:</u> Annual. If foci of SRCC and the patient chose to continue on surveillance: endoscopy every 6 months<br>–<br>Those declining RRTG and having no family history of DGC: Annual endoscopic surveillance + random biopsies.	47, 48, 50, 55,7 1, 73, 96, 101
				<u>CDH1-:</u> Annual. After 2 negative procedures: 2-yearly intervals	
	Breast	Clinical examination +	Age 30	Every 6 months	49

Syndrome	Organ	Method	Start	Frequency	References
		US or mammography, alternately with MRI			
**HLRCC**	Kidney	Renal MRI	Age 10	Annually	74
**LFS**	Whole body	Physical examination	Age 3	Every 3-4 months	24, 32, 46, 76, 77
			Age 16	Annually	
	ACC	Abd and pelvis US + urine and blood test	Age 3 (until age 40)	Every 4 months	
	Brain	MRI	Age 3[46]	Annually	
	Sarcoma	Rapid total body MRI	Age 3 [46]	Annually	
		Abd and pelvis US	Age 18	Every 3-6 months	
	Leukemia-Lymphoma	Blood test	Age 3	Every 4 months	
	Breast	Self-examination	Age 18	Monthly	
		Clinical examination	Age 25	Every 6 months	
		Mammography and MRI	Age 20-25	Annual	
	CRC	Colonoscopy	Age 25[32,46] or 10 years before the earliest CRC in the family	Every 2-3 years	
	Melanoma	Total skin examination	Age 18	Annually	
**PJS**	Whole body	Physical examination	Age 10	Annually	47, 52, 80,85,86
	GIT	US	< 10 years	1-2 years	

Syndrome	Organ	Method	Start	Frequency	References
		Endoscopy	Age 10		
		Colonoscopy	Age 12 [47] or age 25 [52]	2-5 years [52]	
	Pancreas	Abdominal MRI and CA 19-9	Age 18[86]–age 25[47]	Annually	
	Breast	MRI	Age 25	Annually	
		Mammography	Age 30		
	Uterus-Ovary	Cervical smear, TVUS and CA-125, MRI	Age 20 [47], 25-30 years[52]	Annually	
	Testicles	Physical examination + US	Birth	Annually	
**PHTS**	Breast	MRI	From age 25 to age 60	Annually. After age 60, annual mammography. (64 sensitivity, 82 specificity)	90,91
		Mammography	From age 30		
	Uterus	Pelvic exam, TVUS, endometrial biopsy	From age 30 to age 60	Annually	
	CRC	Colonoscopy	From age 40	Every 5 years (or more if indicated by MD or if symptoms)	93
	Thyroid	US	From age 18	Annually (and additionals when indicated)	92

Abbreviations: ACC, adrenocortical carcinoma; BC, breast cancer; Birt Hogg Dube; CRC, colorectal cancer; CT, computerized scan; DGC, diffuse gastric cancer; GIT, gastrointestinal tract; HDGC, hereditary diffuse gastric cancer; HLRCC, hereditary leiomyomatosis and renal cell cancer; HRCT, high resolution computerized scan; MRI, magnetic resonance imaging; MRCP, magnetic resonance cholangiopancreatography; PHTS, *PTEN* hamartoma tumor syndrome; PJS, Peutz-Jeghers syndrome; RRTG, risk reducing total gastrectomy; SRCC, signet ring cell carcinoma; TVUS, transvaginal ultrasound; US, ultrasound.
